# Molecular pathogenesis and novel therapeutic avenues in alpha-fetoprotein-producing gastric cancer

**DOI:** 10.3389/fonc.2026.1814851

**Published:** 2026-04-20

**Authors:** Gaorui Liu, Jinming Zhang, Fujian Ji, Zhongmin Li

**Affiliations:** Department of Gastrointestinal and Colorectal Surgery, China-Japan Union Hospital of Jilin University, Changchun, China

**Keywords:** alpha-fetoprotein-producing gastric cancer, diagnosis, pathogenesis, prognosis, treatment

## Abstract

Alpha-fetoprotein-producing gastric cancer (AFPGC) is a highly malignant subtype of gastric cancer with unique biological characteristics and clinical phenotypes. With the continuous development of molecular biology techniques and diagnostic methods, there has been a deeper understanding of AFPGC in terms of its epidemiological distribution, pathological features, and molecular-level pathogenesis. However, early identification and effective intervention for this type of tumor remain challenging in clinical practice. Current research mainly focuses on discovering potential molecular markers, optimizing diagnostic procedures, and exploring feasible treatment pathways. Despite these efforts, the specific pathogenic mechanism of AFPGC still needs further clarification, and the construction of individualized, multi-modal combined treatment systems also requires further improvement. This article systematically reviews the latest research achievements in the field of AFPGC, aiming to comprehensively sort out the existing knowledge system, provide references for further understanding the complexity of this disease, and promote breakthroughs and innovations in future diagnosis and treatment strategies.

## 1 Introduction

Gastric cancer is one of the most prevalent malignant tumors of the digestive system in China. Globally, it ranks fifth among the most common cancers and fourth among leading causes of cancer-related mortality (1). Alpha-fetoprotein-producing gastric cancer (AFPGC) constitutes a rare subtype of gastric cancer, accounting for 1.3% – 15% of all gastric cancer cases (2–5). This distinct entity was first described by Bourrelie et al. in 1970 (6). For its diagnosis, it is widely accepted that a serum AFP level exceeding 20 ng/mL is indicative of AFPGC (7). With advancements in immunohistochemical (IHC) techniques, some researchers have proposed defining AFPGC based on AFP IHC positivity (8). However, a recent case report documented an instance of gastric cancer with negative AFP IHC staining yet elevated serum AFP levels, which challenges the current diagnostic criteria for AFPGC (9). The current prevailing view holds that AFPGC should be characterized by elevated serum AFP levels or positive AFP IHC staining. Presently, AFPGC is classified into four distinct morphological subtypes: hepatoid, yolk sac tumor-like, fetal gastrointestinal type, and mixed type (10). This classification holds significant implications for prognosis, biological behavior, and therapeutic strategy in AFPGC.

AFPGC exhibits notably more aggressive biological behavior compared to conventional gastric cancer (CGC). A hallmark of this malignancy is its association with a more advanced disease stage at diagnosis (11, 12). Furthermore, AFPGC demonstrates a pronounced predilection for hematogenous dissemination, with the liver being the most frequent and characteristic site of distant metastasis. This metastatic tropism, coupled with its typically higher pathological grade and more invasive growth pattern, contributes to the overall poorer prognosis observed in patients with AFPGC. Specifically, relevant research statistics show that the one-year survival rate of AFPGC patients ranges from 38.7% to 64%, and the five-year survival rate ranges from 4.7% to 41%, which is significantly lower than that of CGC patients (4, 13–15). Serum AFP level has been identified as an independent prognostic factor for gastric cancer (16, 17). Numerous studies have demonstrated that elevated serum AFP levels correlate with unfavorable prognosis and shorter survival outcomes (3, 18). The degree of tumor differentiation in gastric cancer is closely associated with serum AFP levels, with significantly higher AFP concentrations observed in low-differentiated tumors compared to high-differentiated ones (19, 20). Furthermore, the reduction of serum AFP levels has been correlated with chemotherapy response and survival rates. Although serum AFP alone cannot be definitively linked to survival outcomes, studies have shown that a decrease in AFP levels is significantly associated with improved prognosis (21, 22), highlighting the importance of real-time AFP monitoring during AFPGC treatment. Additionally, tracking changes in AFP levels following first-line chemotherapy may provide insights into tumor behavior and guide subsequent therapeutic decisions.

Overall, AFPGC is a rare but aggressive subtype of gastric cancer. Despite its clinical significance, the diagnosis and management of AFPGC remain challenging due to unclear diagnostic criteria and the lack of standardized treatment protocols. In addition, the biological characteristics and molecular mechanisms underlying this disease are not fully understood, leading to variability in clinical practice and outcomes. This review comprehensively summarizes recent advances in research on AFPGC, covering its epidemiology, pathogenesis, diagnosis, clinical features, treatment strategies, and prognosis. Special emphasis is placed on the latest developments in understanding the molecular mechanisms underlying AFPGC, as well as emerging therapeutic approaches. This synthesis of knowledge provides a basis for developing evidence-based clinical practice guidelines to advance the understanding of AFPGC and ultimately improve outcomes for this aggressive cancer.

## 2 Epidemiology

The reported global incidence of AFPGC is relatively low, ranging from 1.3% to 15%. Notably, the incidence in China reaches approximately 6.63%, which is significantly higher compared to other regions (4). Current evidence indicates that AFPGC is associated with a particularly poor prognosis and elevated mortality rates (23). Numerous studies have investigated the survival outcomes of AFPGC patients and observed significantly poorer one-year, three-year, and five-year survival rates compared to their AFP-negative counterparts. In these studies, AFP‑negative status was generally defined by serum AFP levels below the diagnostic cutoff of 20 ng/mL. Notably, the five-year mortality rate among AFPGC patients is reportedly approximately 72%. Furthermore, elevated AFP levels have been associated with a 68% increased risk of mortality (24). This malignancy demonstrates a predilection for male patients and predominantly affects middle-aged to elderly populations. To date, there remains a scarcity of authoritative long-term epidemiological data to definitively establish the temporal trends in incidence rates. However, advancements in serum AFP testing and IHC techniques have enhanced diagnostic accuracy, leading to increased identification of previously undetected cases and potentially contributing to an apparent rise in detection rates.

## 3 Pathogenesis

**Figure 1:**
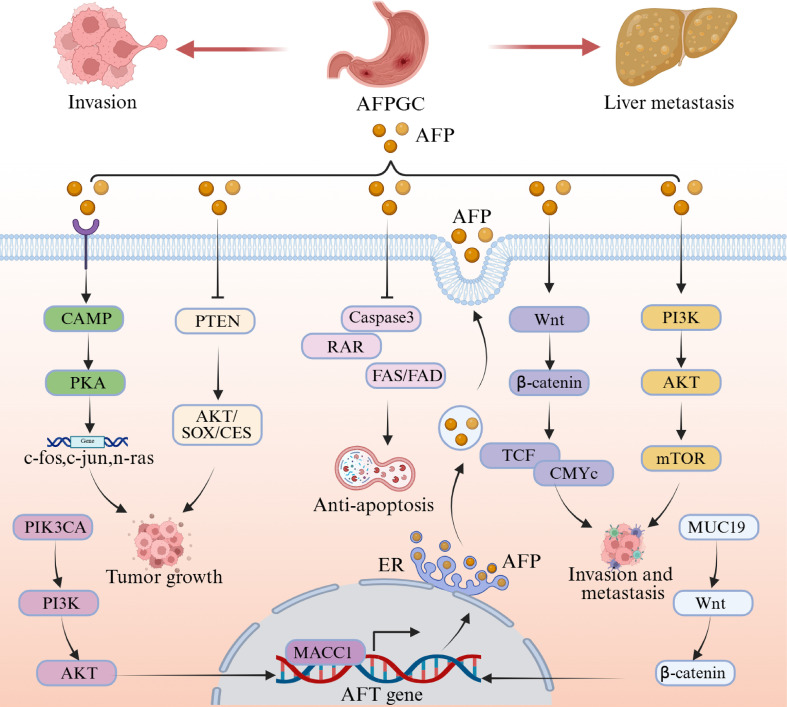
Interplay between AFP and oncogenic signaling networks in AFPGC. The illustration depicts how aberrant AFP expression both drives and is sustained by the activation of multiple pathways (e.g., Wnt/β-catenin, PI3K/AKT, cAMP-PKA), collectively enhancing proliferation, invasion, and survival of gastric cancer cells.

The molecular pathogenesis of AFPGC has emerged as a key focus of recent research. The onset and progression of AFPGC involve intricate regulatory mechanisms encompassing multiple signaling pathways and molecular networks. High levels of AFP are closely associated with the aberrant activation of several key signaling pathways, which play significant roles in tumor development. Activation of the Wnt/β-catenin pathway has been shown to promote AFP expression, while AFP itself can reciprocally enhance Wnt signaling, collectively reinforcing tumor cell proliferation and invasive capacity (25, 26). Similarly, PIK3CA upregulates AFP levels via activation of the PI3K/AKT axis, concurrently stimulating tumor growth and suppressing apoptosis (27, 28). A pathway enrichment analysis revealed that numerous critical signaling pathways were significantly enriched in AFPGC patients, including those involved in disease-related signaling processes, receptor tyrosine kinase pathways, intracellular second messenger systems, PI3K-AKT signaling, MAPK signaling cascades, nuclear receptor-mediated pathways, FLT3 signaling, MAPK1/MAPK3 signaling, and estrogen receptor-mediated signal transduction (28). These signaling pathways were found to be strongly linked to critical processes such as tumor cell proliferation, invasion, and metastasis. AFP overexpression contributes to oncogenesis by interfering with apoptotic pathways, such as through inhibition of caspase-3 and RAR-mediated pro-apoptotic signaling (29, 30). Additionally, AFP activates the cAMP-PKA signaling cascade, inducing oncogene transcription and thereby accelerating tumor cell proliferation (31). Moreover, AFP facilitates tumor progression by downregulating PTEN expression and modulating the AKT1/SOX5/CES1 signaling cascade (32). To provide a systematic overview of these interconnected signaling networks in AFPGC, we have integrated current findings into a schematic diagram presented in **Figure 1**.

Recent investigations have demonstrated that AFP expression is tightly regulated by an array of specific molecular determinants. Mechanistic studies have shown that AFP promoter reactivation, driven by either the inhibition of enhancer elements or the removal of silencer regions, leads to significantly elevated AFP expression levels. Notably, miR-122-5p has been identified as a critical regulator in this context, with its expression being markedly upregulated in AFPGC compared to CGC (33). This microRNA plays a pivotal role in promoting tumor progression through its direct interaction with FOXO3, thereby suppressing apoptosis (34). Furthermore, loss of ATBF1 expression is associated with increased AFP expression levels (35). Additional research has identified several other key regulatory molecules, including HBP1, ZBTB1, HNF4A, CEBP-β, and FOXA2, which collectively modulate AFP expression through distinct mechanisms (36–40). These findings collectively highlight the intricate molecular networks governing AFP expression and provide valuable insights into the underlying mechanisms of AFPGC. Moreover, they underscore the potential for developing novel therapeutic intervention strategies targeting these specific regulatory pathways in this aggressive subtype of gastric cancer.

Elevated AFP expression is a key prognostic indicator associated with poor clinical outcomes in AFPGC patients. Recent studies have highlighted angiopoietin-like protein 6 (ANGPTL6) as a critical factor in this aggressive malignancy, wherein compelling evidence demonstrates that ANGPTL6 expression is markedly elevated in AFP-positive gastric cancer cells relative to CGC cells (41). Notably, ANGPTL6 expression levels exhibit a significant positive correlation with AFP levels, highlighting its potential role as a therapeutic target. ANGPTL6 promotes tumor angiogenesis and progression by activating the ERK1/2 and AKT signaling pathways, which may underpin the highly malignant phenotype observed in AFPGC. In addition to these findings, hepatocyte growth factor (HGF) and its receptor c-Met have been implicated in the acquisition of invasive phenotypes and distant metastasis in tumor cells. Research indicates that the high incidence of liver metastasis in AFPGC patients may be closely linked to the aberrant overexpression of c-Met (42). Importantly, high levels of AFP have been shown to upregulate c-Met expression, further emphasizing the potential role of this pathway in disease progression. Moreover, metastasis-associated in colon cancer 1 (MACC1), a key upstream transcriptional regulator of c-Met, is frequently overexpressed in gastric cancer and has been significantly associated with poor patient prognosis (43). These findings collectively underscore the critical role of the HGF/c-Met signaling axis, modulated by both AFP and MACC1, in driving the aggressive clinical behavior of AFPGC. Elucidating these molecular mechanisms provides valuable insights into potential therapeutic targets for this highly malignant cancer subtype.

## 4 Diagnosis

### 4.1 Serological diagnosis

AFP is a single-chain glycoprotein structurally analogous to albumin, primarily synthesized in the liver and yolk sac during fetal development (44). AFP levels are notably elevated during the neonatal period but decrease significantly in adult serum. However, a marked increase in AFP expression is observed when hepatocytes undergo malignant transformation. The regulation of AFP expression predominantly occurs at the transcriptional level, with liver-enriched nuclear factor playing a critical role in modulating its transcriptional activity in fetal hepatic tissue (45). Clinically, serum AFP represents the most significant tumor marker for the diagnosis of primary hepatocellular carcinoma (46, 47). Notably, elevated AFP levels are associated with various malignancies of the digestive system, including hepatocellular carcinoma, gastric cancer, colorectal cancer, and cholangiocarcinoma (48).

In the seminal case report by Bourreille et al., AFPGC was first defined as a gastric cancer subtype associated with elevated AFP levels. While a serum AFP level >20 ng/mL is widely regarded as having clinical diagnostic significance, no universally accepted and evidence-based threshold has been established. Research definitions vary considerably, with some studies employing cut-off values such as >160 ng/mL (21), while others have used thresholds of 270 ng/mL, 300 ng/mL, or even 1000 ng/mL to identify cases with markedly elevated AFP (3, 16). Given the current lack of sufficient evidence to support a standardized threshold, future large−scale, prospective, multicenter studies are warranted to validate an optimal cutoff value for routine clinical use.

### 4.2 Imaging diagnosis

Imaging examination plays a crucial role in the diagnosis, staging, and therapeutic evaluation of gastric cancer. Modalities such as ultrasonography, computed tomography (CT), and magnetic resonance imaging (MRI) provide detailed visualization of tumor size, location, and anatomical relationships with adjacent structures. CT imaging, in particular, is valuable for assessing gastric wall thickening, local invasion, and distant metastases (49). Studies have confirmed that CT can effectively stage gastric cancer and inform surgical strategy (50, 51). Compared with CGC, AFPGC often exhibits distinct imaging features, including marked gastric wall thickening and ulceration on CT, with most patients presenting at an advanced stage at initial diagnosis (51). Accurate imaging differentiation is therefore critical for determining the optimal treatment approach. Complementary measures, such as timely liver biopsy and close monitoring of serum AFP levels, are also important in clinical management (52). MRI provides superior soft-tissue resolution and contrast, significantly enhancing the characterization and localization of lesions. Notably, in the context of gastric cancer with liver metastases, MRI demonstrates exceptional diagnostic accuracy in preoperative T, N, and M staging, thereby substantially improving overall staging precision (53). This modality plays a pivotal role in optimizing treatment planning and prognosis assessment for patients with AFPGC and suspected hepatic metastases.

Through the synergistic combination of positron emission tomography (PET) and CT, PET-CT integrates functional metabolic imaging with precise anatomical localization. This hybrid modality enables accurate detection of subtle metastatic lesions throughout the body with high sensitivity, while also providing significant diagnostic utility in distinguishing primary liver cancer from hepatic metastases. Lesions of primary liver cancer are characteristically associated with intense fluorodeoxyglucose (FDG) uptake and represent the predominant space-occupying lesions within the liver. In contrast, hepatic metastases related to advanced gastric cancers often exhibit hypermetabolic features; however, these are typically accompanied by robust FDG uptake in the primary gastric tumor. This distinct dual metabolic signature facilitates effective discrimination between primary liver malignancies and metastatic deposits of gastric origin (54).

### 4.3 Endoscopic diagnosis

Endoscopy plays a fundamental role in the diagnosis of gastric cancer, enabling early lesion detection, differentiation between benign and malignant ulcers, and precise evaluation of tumor type and infiltration depth (55). In the context of AFPGC, the diagnostic value of endoscopy is particularly critical. Endoscopic ultrasound further enhances diagnostic accuracy by providing detailed assessment of tumor invasion depth and local extent, which is essential for precise staging and the formulation of individualized, evidence-based treatment strategies (56). Accurate diagnosis and staging are crucial for AFPGC, as they directly inform treatment decisions and prognostic evaluation.

In addition to its diagnostic role, endoscopy also offers therapeutic applications. For superficial AFPGC confined to the mucosal layer without lymph node metastasis, endoscopic submucosal dissection provides a minimally invasive, curative option that enables complete tumor resection while preserving organ function and minimizing surgical trauma (57, 58). For patients with advanced disease complicated by gastrointestinal bleeding or luminal obstruction, endoscopy serves a palliative function, facilitating hemostasis and enteral nutrition tube placement to stabilize nutritional status and improve quality of life.

### 4.4 Pathological diagnosis

Histologically, AFPGC often exhibits clear tumor cells that morphologically resemble fetal intestinal epithelium, a phenotype attributed to tumor cell transdifferentiation (59). Immunohistochemically, these tumor cells typically show positivity for AFP, Claudin-6, glypican-3, cytokeratin 19, and c-Met. In contrast, the expression of hepatocyte-specific markers, such as hepatocyte membrane antigen and arginase-1, can be variable or absent (5, 60–62). Distinguishing AFPGC from hepatocellular carcinoma can be particularly challenging in cases of metastatic disease with an unknown primary. Emerging evidence suggests that SALL4 positivity is a highly discriminatory marker favoring a diagnosis of AFPGC over hepatocellular carcinoma (63). Additionally, SMARCB1 has been proposed as a valuable biomarker to aid in differentiating metastatic AFPGC from hepatocellular carcinoma (64).

Studies have demonstrated that distinct subtypes of gastric cancer can be characterized through cytological and histological analyses to determine tumor type and grade (65). The most widely adopted pathological classification system, proposed by Motoyama et al., is based on reproducible histological patterns. It categorizes AFPGC into four distinct subtypes: the hepatoid type, which resembles hepatocellular carcinoma; the yolk sac tumor-like type, which mimics yolk sac tumors; the fetal gastrointestinal type, characterized by a fetal gut-like appearance; and the mixed type, which exhibits a combination of the above patterns (66). This classification was subsequently validated through the establishment of heterotransplantation models (67). These four subtypes constitute the main histological classification system for AFPGC.

Notably, the hepatoid type represents a unique and highly malignant pathological subtype and is considered one of the major components of AFPGC (13). Previous studies have demonstrated that this subtype is characterized by poor differentiation, a high frequency of lymphatic and vascular invasion, and the most consistent AFP immunoreactivity. In contrast, the yolk sac tumor-like type may exhibit the most aggressive behavior; these tumors are predominantly poorly differentiated and diffuse according to the Lauren classification, with elevated carcinoembryonic antigen (CEA) expression being the most common finding (68). Another study further confirmed that the yolk sac tumor-like type is associated with the poorest prognosis, although no significant differences in overall survival (OS) were observed among the four subtypes (10, 68). Notably, the reported proportions of these four subtypes vary across studies, which may be attributable to limited sample sizes. These findings underscore the need for large-scale, multicenter studies to better characterize the histological classification of AFPGC in the future.

Kinjo et al. proposed an alternative classification system based on histological patterns, categorizing AFPGC into four subtypes: common adenocarcinoma type, enteroblastic type, hepatoid type, and yolk sac tumor type (69). According to this model, many AFPGC cases originate as common adenocarcinoma within the mucosa and subsequently acquire enteroblastic or hepatoid differentiation during tumor infiltration and proliferation, thereby gaining the capacity for AFP production. This evolutionary process provides valuable insights into the mechanisms underlying tumor progression and holds significant implications for the future refinement of histological subtyping in AFPGC.

## 5 Clinical characteristics

Most patients with AFPGC present with initial symptoms that are nonspecific and similar to those of CGC, including acid reflux, belching, and irregular upper abdominal pain. AFPGC is associated with a significantly higher incidence of liver metastasis, lymph node metastasis, venous invasion, and perineural invasion compared to gastric cancers with negative serum AFP levels (70). This tumor predominantly arises in the gastric antrum and is most frequently diagnosed at an advanced stage, with stage III and IV disease accounting for approximately 73.3% to 90.2% of cases (10, 15, 22). The rate of lymph node metastasis in AFPGC is markedly elevated relative to CGC, with involvement primarily concentrated in the N2 and N3 stages (14). Liver metastasis represents the most characteristic form of distant spread in AFPGC (70). Studies report liver metastasis rates as high as 45.4%, with approximately 29.27% of patients already exhibiting hepatic involvement at the time of initial diagnosis (71).

## 6 Therapeutic strategies

### 6.1 Surgery

Radical surgical resection remains a cornerstone of curative treatment for gastric cancer. Within the specific subgroup of AFPGC, surgery is still considered the most effective therapeutic option for early-stage disease (72). Despite its aggressive biology, patients with AFPGC who undergo surgical resection experience significantly improved long-term survival and often exhibit a marked postoperative decline in serum AFP levels (73). A retrospective study analyzing 465 patients who underwent gastric cancer surgery revealed that the three-year and five-year OS rates among the 24 AFPGC patients were 85.2% and 75.7%, respectively (74). For non-AFPGC patients during the same period, these figures were 79.6% and 77.7%. Log-rank test analysis indicated no statistically significant difference in OS between AFPGC and non-AFPGC patients. These findings underscore the critical importance of early diagnosis and timely surgical intervention in improving patient outcomes when surgical criteria are met, highlighting the potential benefits of aggressive management strategies for this patient population.

For many patients, perioperative strategies, including neoadjuvant and adjuvant therapies, may be necessary to mitigate recurrence risk. Thus, timely surgical intervention is crucial for improving outcomes in AFPGC. However, the highly malignant nature of AFPGC often leads to diagnosis at an advanced stage, with many patients presenting with synchronous liver metastasis, thereby precluding early curative resection. Currently, no clear consensus exists regarding the optimal management of AFPGC-associated liver metastases or the precise surgical indications for such lesions. While one study reported prolonged survival following resection of hepatic metastases in AFPGC patients (75), insufficient evidence is available to define the definitive role of liver resection within the overall treatment paradigm. Consequently, future studies and larger-scale reports are warranted to evaluate the efficacy and timing of hepatic resection in the setting of AFPGC with liver metastasis.

### 6.2 Chemotherapy

Chemotherapy remains a cornerstone in the management of gastric cancer. Traditional regimens such as XELOX (capecitabine plus oxaliplatin) and SOX (S-1 plus oxaliplatin) continue to serve as standard first-line options for advanced disease [57]. However, AFPGC appears to exhibit broad resistance to conventional cytotoxic agents, a key contributor to its challenging treatment and poor prognosis (4, 76). Emerging evidence suggests that resistance mechanisms in AFPGC may involve dysregulated signaling pathways. For instance, activation of the mammalian target of rapamycin (mTOR) pathway has been implicated in cisplatin resistance, indicating that combining mTOR inhibitors such as rapamycin with cisplatin could represent a promising therapeutic strategy for AFPGC (77). Furthermore, sensitivity to cytotoxic chemotherapy is believed to be influenced by the expression of solute carrier transporters. Investigating the expression profiles of these transporters in AFPGC is therefore essential for developing more effective and personalized chemotherapy regimens in the future (78).

### 6.3 Targeted therapy

Individualized targeted therapy has emerged as a key focus in oncology research. With the continuous advancement of targeted therapy, significant improvements in gastric cancer treatment have been observed, particularly in human epidermal growth factor receptor 2 (HER2)-positive patients, where HER2-targeted therapies have demonstrated remarkable clinical outcomes (79, 80). Studies have consistently shown that targeted therapy exhibits excellent clinical efficacy in treating HER2 positive patients, with all treated patients achieving long-term OS and relapse-free survival (RFS) (81). Notably, partial overexpression of HER2 has been identified in AFPGC, underscoring the potential role of targeted therapy in the combined treatment regimen for AFPGC (79, 82). Furthermore, vascular endothelial growth factor C (VEGF-C) exhibits higher expression levels in AFPGC compared to AFP-negative gastric cancers at the same stage (83). A study highlights the efficacy and safety of the anti-angiogenic drug apatinib in patients with AFPGC, suggesting that anti-angiogenic therapy may represent a promising strategy for this rare subtype of gastric cancer (84). The results demonstrated that apatinib treatment yielded a disease control rate (DCR) of 70% and a median progression-free survival (PFS) of 3.5 months, along with a median OS of 4.5 months. Notably, AFPGC patients without elevated CEA levels achieved a median OS of 30.8 months. These results indicate that apatinib exhibits promising efficacy in patients with advanced AFPGC. Additionally, research indicates that the combination of the anti-angiogenic agent ramucirumab with paclitaxel has shown potential in second-line treatment of AFPGC (85). This study found that the combination therapy regimen resulted in a higher response rate among AFPGC patients compared to non-AFPGC patients (39.1% vs. 24.8%). Specifically, the median PFS was 5.5 months in the AFPGC cohort versus 4.0 months in the non-AFPGC cohort, and the median OS was 10.7 months versus 9.2 months, respectively.

Claudin18.2 has been identified as a potential therapeutic target for gastric cancer (86, 87). However, studies reveal that its expression is significantly downregulated in AFPGC, posing additional challenges in treating this aggressive tumor subtype (88). Recent investigations have discovered that ANGPTL6 promotes tumor angiogenesis by activating the ERK1/2 and AKT pathways, positioning it as a potential therapeutic target (41). Its inhibitors have demonstrated inhibitory effects on tumor growth in preclinical animal models, though further clinical validation is still required. Despite these advances, challenges such as resistance to chemotherapy and targeted therapies persist. Therefore, further exploration of novel treatment options and combination regimens is essential to enhance patient survival outcomes.

### 6.4 Immunotherapy

The application of immunotherapy in AFPGC has garnered increasing attention in recent years. Research has revealed that AFPGC employs multiple immune evasion mechanisms, particularly through the upregulation of HLA-G expression and the lack of HLA-I molecules, which collectively provide promising directions for targeted intervention in immunotherapy (89). Currently, immunotherapy for AFPGC has demonstrated remarkable efficacy. To improve the poor prognosis of AFPGC, several clinical trials are evaluating the efficacy of combination therapies that incorporate immunotherapy, targeted therapy, or chemotherapy, aiming to develop more effective treatment strategies for this aggressive subtype (**Table 1**). Recent evidence demonstrates that combining immune checkpoint inhibitors (such as programmed death-1 (PD-1) antibodies) with chemotherapy significantly improves PFS and OS in patients with AFPGC (90, 91). A clinical study evaluated the safety and efficacy of a quadruple regimen comprising GLS-010 (an anti-PD-1 antibody), lenvatinib, and XELOX chemotherapy in patients with AFP-elevated and HER2-negative gastric cancer (90). The results showed an objective response rate (ORR) of 33.3% and a DCR of 100%, with a median PFS of 7.67 months and a median OS of 13.17 months. A multicenter, single-arm phase II trial evaluated the antitumor activity and safety of camrelizumab (an anti-PD-1 antibody), apatinib, and SOX in 35 enrolled patients with AFPGC (92). This regimen achieved an ORR of 66.7% and a DCR of 88.9%, with a median PFS of 7.8 months and a median OS of 18.0 months. These promising clinical outcomes underscore the significant therapeutic potential of immunotherapy in AFPGC, offering new hope for improving survival and quality of life in this patient population. A case report demonstrated that a patient with AFPGC successfully achieved conversion therapy and became eligible for surgical resection following treatment with an immunotherapy−chemotherapy combination regimen, with notable therapeutic responses observed both pre− and postoperatively (93). This finding further supports the efficacy of immunotherapy−based combination strategies in the management of AFPGC (93, 94).

**Table 1:** Clinical trials of AFPGC.

ClinicalTrials.gov identifier	Treatment	Start year	Phase	Number of patients	Region/country	Status
NCT04006821	Camrelizumab and apatinib mesylate	2019	II	30	Shenyang, China	Unknown
NCT06383559	XELOX, lenvatinib, and sintilimab	2023	II	39	Tianjin, China	Recruiting
NCT05221775	XELOX, GLS-010, and lenvatinib	2021	I	18	Tianjin, China	Unknown
NCT04609176	SOX, camrelizumab, and apatinib	2020	II	64	Beijing, China	Unknown
NCT06427941	BGB-B2033 and tislelizumab	2024	I	140	United States, China, New Zealand, and South Korea	Recruiting
NCT07289997	Iparomlimab, tuvonralimab, apatinib, and irinotecan hydrochloride	2025	II	39	Shijiazhuang, China	Recruiting

Furthermore, a study identified that the nutritional status of patients is closely correlated with the effectiveness of immunotherapy, suggesting that optimal nutritional health may improve treatment outcomes (95). Therefore, clinicians should tailor therapeutic regimens to the specific conditions and needs of individual patients in order to maximize treatment efficacy and enhance survival rates. The precise and accurate assessment of immunotherapy efficacy in patients with AFPGC represents a critical research priority in contemporary oncology. Recent investigations have emphasized the importance of developing innovative and reliable tools to optimize prognostication, treatment planning, and therapeutic decision-making in clinical oncology. To address this need, one study utilized a rigorous multivariate analysis framework to develop and validate the ANLiM score, an innovative predictive model specifically designed for patients with AFPGC (96). The ANLiM score is composed of two key prognostic factors: serum AFP levels and the neutrophil-to-lymphocyte ratio, both of which were identified as significant independent predictors through comprehensive statistical analysis. This novel index not only demonstrates high accuracy in predicting liver metastasis but also provides valuable insights into forecasting patient responses to immunotherapy, thereby offering a powerful tool for personalized treatment strategy. This advancement holds profound implications for optimizing immunotherapy assessment strategies, offering a valuable tool for personalized treatment approaches and ultimately improving clinical outcomes in this patient population.

With the expanding landscape of therapeutic options for gastric cancer, multi-disciplinary teams (MDTs) have become increasingly pivotal in delivering personalized treatment strategies. Its principal advantage lies in the accurate staging of cancer and the formulation of integrated treatment plans, which may combine neoadjuvant therapy, surgery, targeted agents, and immunotherapy. This collaborative decision-making process has been shown to enhance therapeutic precision and improve patient survival. Evidence underscores the significant role of MDTs in the management of gastrointestinal tumors (97). Consequently, leveraging multidisciplinary consultation is of critical importance for ensuring precise diagnosis and tailored, individualized treatment for patients with AFPGC. In recent years, artificial intelligence (AI) has experienced rapid advancement and integration into healthcare. A retrospective study has demonstrated that AI-driven diagnostic and therapeutic recommendations show substantial alignment with those generated by the MDT model in gastric cancer management (98). This concordance further validates the significant and growing role of AI in supporting and potentially augmenting clinical decision-making for AFPGC.

### 6.5 Other treatment strategies

Radiotherapy is primarily utilized in the management of AFPGC for the local control of metastatic lesions. Studies indicate that for unresectable metastases, such as recurrent para-aortic lymph node involvement, radiotherapy can effectively control disease progression and extend patient survival (99). Radiofrequency ablation (RFA), a minimally invasive local therapy, is often employed for AFPGC-related liver metastases. In cases of solitary and non-resectable hepatic lesions, RFA achieves tumor cell destruction through thermal energy, thereby improving quality of life in advanced-stage patients. Furthermore, transcatheter arterial infusion chemotherapy (TAI) represents a key interventional approach for liver metastases in AFPGC. This modality enables the direct delivery of chemotherapeutic agents to metastatic foci via the hepatic artery, thereby enhancing local drug concentration and antitumor efficacy (100). Clinical reports have demonstrated that TAI combined with systemic chemotherapy results in a significant reduction in serum AFP levels, with follow-up imaging showing complete remission in some patients (101). These findings suggest that such combined locoregional and systemic strategies hold potential for controlling metastatic disease and improving therapeutic outcomes in AFPGC.

## 7 Prognosis

Compared to patients with CGC, those with AFPGC exhibit a markedly poorer overall prognosis, with the majority diagnosed at an advanced stage (11, 12, 23). The incidences of liver metastasis, lymph node metastasis, venous invasion, and perineural invasion are significantly higher in AFPGC than in CGC (14, 70). We have compiled relevant research data pertaining to the survival outcomes of AFPGC patients, as summarized in **Table 2**. Our analysis revealed that the survival rates for AFPGC patients at each tumor stage were significantly lower than those for CGC patients, underscoring their poorer prognosis. Serum AFP levels represent an independent prognostic factor for gastric cancer (16, 17). Furthermore, a correlation exists between gastric cancer differentiation status and serum AFP levels, with significantly higher AFP concentrations observed in poorly differentiated tumors compared to well-differentiated cases (10, 19, 20). A decrease in serum AFP levels has been linked to both chemotherapy response and survival outcomes. Although serum AFP alone cannot be definitively correlated with survival, reductions in AFP levels are significantly associated with improved prognosis (21, 22), highlighting the importance of real-time AFP monitoring during AFPGC treatment. Additionally, tracking changes in AFP following first-line chemotherapy may provide insights into tumor behavior and inform subsequent therapeutic strategies.

**Table 2:** Comparison of survival rates between AFPGC and CGC.

1-year OS (%)		3-year OS (%)		5-year OS (%)		P	Reference
AFPGC	CGC	AFPGC	CGC	AFPGC	CGC		
38.7	71.3	11.6	57.8	11.6	52.8	<0.001	(4)
–	–	–	–	28.4	45.0	<0.001	(14)
53	95	35	57	28	38	0	(23)
64	95	47	57	41	38	0	(13)
46.7	75.2	28.9	53.4	17.8	45.8	<0.001	(12)
62.7	84.6	27.5	55.4	4.7	16.5	<0.001	(15)

In patients with AFPGC, the occurrence of liver metastasis is notably high. Research has shown that liver metastasis is considered an independent adverse prognostic factor for AFPGC (23). Moreover, the prognosis of AFPGC is influenced by a variety of factors. Key prognostic factors include vascular invasion, lymph node metastasis, serosal invasion, pathological staging, and preoperative serum CEA levels, among others. Through a systematic review of the literature, we have carefully selected crucial prognostic indicators from numerous studies and summarized them in **Table 3**. The results indicate that, compared to CGC, AFPGC is characterized by a poor prognosis, with distinctive features such as a high rate of vascular invasion, a high lymph node metastasis rate, and a significantly elevated liver metastasis rate.

**Table 3:** Comparison of prognostic characteristics between AFPGC and CGC.

Liver metastasis (%)		Vessel invasion (%)		Lymphatic metastasis (%)		Year	Reference
AFPGC	CGC	AFPGC	CGC	AFPGC	CGC		
63	9	48.1	46.7	85.2	45.7	2002	(14)
60.6	11.5	63.5	49.5	81.7	70.2	2010	(23)
14.3	3.6	22.9	7.9	68.6	48.9	2011	(11)
49.2	11.5	54.2	49.5	76.3	70.2	2012	(13)
91.4	60.7	17.2	3.8	27.6	4.4	2014	(12)
–	–	66.6	0.4	33.3	2.3	2016	(108)

## 8 Conclusion and future prospects

AFPGC represents a distinct subtype of gastric cancer with unique biological behavior and clinical features. Although progress has been made in characterizing its clinicopathological characteristics, the underlying molecular mechanisms and optimal treatment strategies remain incompletely understood. Most patients are diagnosed at an advanced stage, contributing to the poor prognosis associated with this disease. Consequently, the precise identification and validation of molecular markers constitute a critical priority for future research. As the defining biomarker of AFPGC, AFP continues to warrant further investigation regarding its clinical utility and molecular mechanisms. Although AFP is a hallmark of this entity, the precise relationship between its expression levels and disease progression, metastasis, and prognosis remains to be fully elucidated. Recent findings suggest that AFP stabilizes the oncoprotein c−MYC through interaction with heat shock protein 90 (102). In addition, the clinical significance of immune-related biomarkers, particularly programmed death-ligand 1 and microsatellite instability, should be explored in depth to improve treatment efficacy and prognostic accuracy in AFPGC.

Beyond established markers, the identification of driver gene aberrations offers important opportunities for targeted therapy. Integrative analyses of potential target genes have identified ERBB2 and CCNE1 as two frequently altered genes in AFPGC, with their corresponding therapeutic targets already validated. In cases with co−amplification of ERBB2 and CCNE1, combined inhibition of both targets represents a promising precision treatment strategy (3). Moreover, emerging markers warrant attention. Dickkopf−1 (DKK1) has been shown to promote cell proliferation and migration, regulate AFP expression, and drive malignant phenotypes, positioning it as a potential diagnostic biomarker and therapeutic target (103). Notably, DKK1 is also implicated in immune evasion and may attenuate the efficacy of immunotherapy in gastric cancer (104). Looking forward, multi−omics strategies integrating genomics, transcriptomics, and proteomics hold great promise for the discovery of more reliable and specific molecular markers. Such integrative analyses are expected to enhance early diagnosis and improve prognostic assessment for patients with AFPGC.

Currently, antibody-drug conjugates (ADCs) have demonstrated significant potential. Although dedicated studies on ADC monotherapy for AFPGC are limited, combination therapies incorporating ADCs with other treatment modalities offer innovative approaches to managing the disease. For instance, representative ADC drugs targeting HER2-positive gastric cancer, such as trastuzumab deruxtecan and RC48, have been explored in clinical settings (105, 106). Furthermore, ongoing investigations into ADCs targeting glypican-3 provide additional directions for advancing precise treatment strategies in AFPGC (107).

Moreover, the lack of suitable animal models has significantly hindered mechanistic studies and therapeutic development for AFPGC. A promising strategy to address this gap is the development of patient-derived xenograft (PDX) models, which involve transplanting fresh patient tumor tissue into immunodeficient mice. PDX models are particularly valuable as they retain key molecular, genetic, and histopathological features of the original human tumor, thereby providing a biologically relevant platform for studying tumor progression, metastasis, and therapeutic response (3). Establishing reliable PDX models that accurately recapitulate the clinical and molecular profile of AFPGC is therefore critical for elucidating disease pathogenesis, conducting preclinical drug screening, and evaluating novel therapeutic strategies.

Finally, the majority of studies investigating the clinical features of AFPGC and related topics are based on single−institution, retrospective designs. Such studies are inherently susceptible to selection bias, limited by relatively small sample sizes, and often exhibit heterogeneity in follow−up duration and treatment protocols. These factors collectively compromise the robustness and generalizability of their findings. Consequently, the prognostic models and candidate biomarkers currently reported lack validation in large, diverse, multicenter cohorts, leaving their real−world clinical utility and predictive accuracy largely unconfirmed. To address these limitations, future research should prioritize large−scale, prospective, multicenter studies with standardized follow−up. Such efforts are essential to rigorously validate the clinical relevance of prognostic indicators and molecular biomarkers, thereby providing high−quality evidence to inform risk−stratified management and personalized therapeutic strategies for patients with AFPGC.

In conclusion, addressing the unique challenges posed by AFPGC requires a multidisciplinary approach that integrates basic science, translational research, and clinical trials. The development of tailored therapies, combined with a deeper understanding of molecular mechanisms, will be essential for improving patient outcomes in this complex malignancy. By leveraging cutting-edge technologies, exploring novel therapeutic targets, and fostering international collaboration, researchers can unlock the full therapeutic potential of AFPGC treatment, ultimately paving the way for more effective and personalized care for patients worldwide.
